# Discontinuation and nonpublication analysis of chronic pain randomized controlled trials

**DOI:** 10.1097/PR9.0000000000001069

**Published:** 2023-04-04

**Authors:** Samuel M. Jacobsen, Ty Moore, Alexander Douglas, Drew Lester, Austin L. Johnson, Matt Vassar

**Affiliations:** Office of Medical Student Research, Oklahoma State University Center for Health Sciences, Tulsa, OK, USA

**Keywords:** Chronic pain, Research waste, Discontinuation, Nonpublication

## Abstract

**Introduction:**

The primary objective of this cross-sectional analysis is to evaluate rates of discontinuation and nonpublication of Randomized controlled trials (RCTs) of therapeutic interventions to treat chronic pain.

**Methods:**

Using ClinicalTrials.gov, a sample was obtained which included clinical trials pertaining to chronic pain. Trials were analyzed for publication status and completion status of each trial. If information was unavailable on the trial registry database, or could not be allocated through a systematic search, the corresponding trialist was contacted and data points were gathered.

**Results:**

In our final analysis of the 408 RCTs, we found that 281 (68.9%) were published in a peer-reviewed journal and 127 (31.1%) were unpublished trials. Of 112 discontinued trials, 59 (52.7%) reached publication. In addition, 221 of 296 completed trials (74.7%) were published, and 75 (25.3%) remained unpublished after trial completion. The most common listed reason for trial discontinuation was administrative recommendations (41 of 71 trials [57.7%]), while not receiving an email reply to our standardized email from the corresponding trialist was the most common result for trial nonpublication (49 of 88 trials [55.7%]). Clinical trials funded by nonindustry sponsors were more likely to reach publication than industry-funded clinical trials (unadjusted odds ratio 1.86 [95% CI, 1.18–2.95]; adjusted odds ratio 3.01 [95% CI, 1.76–5.14]).

**Conclusion:**

The rate of discontinuation of RCTs involving patients with chronic pain is concerning. Chronic pain affects many patients; thus, the importance of having quality data from clinical trials cannot be overstated. Our study indicates that chronic pain RCTs are frequently discontinued and their findings often go unpublished — all of which could provide crucial information to providers and patients regarding the treatment of chronic pain. We offer suggestions to enhance chronic pain RCT completion, thereby reducing the waste of resources in chronic pain research.

## Abstract 2

Supplemental Digital Content is Available in the Text.

## 1. Introduction

Randomized controlled trials (RCTs) are pivotal for the development of evidence-based medicine^14^ and the progression of patient care for managing chronic pain^32^ — a condition affecting 50 million U.S. adults and costing more than $560 billion annually.^8,18^ Some of the objectives of RCTs are to evaluate efficacy, determine survival, estimate complication rates, and predict responses.^37^ For example, an RCT conducted on 240 patients with arthritis demonstrated that the initiation of opioids for moderate-to-severe chronic back pain, hip, or knee osteoarthritis pain was not superior to nonopioid therapies for improving pain-related function.^22^ Because of the importance of clinical trials, research waste of any kind is an unwise use of scarce scientific resources and hinders the progression of science.^26,36^

One form of research waste is clinical trial discontinuation. Many RCTs are left unfinished without any known reasons or results, which prevents the further advancement of clinical medicine. As stated by the Declaration of Helsinki, the discontinuation of RCTs for unethical reasons (ie, financial or personal) can jeopardize patient safety and contribute to further research waste.^12,35^ Therefore, the only ethical reasons to discontinue an RCT would be related to participant safety, feasibility, or trial efficacy.^9,24,27^ Despite these standards, RCTs are often discontinued for preventable reasons^3^; for example, one systematic review found 76% of RCTs were discontinued for poor recruitment, a prominent preventable reason.^2,20^ All aspects of RCT initiation and implementation need to be thoroughly considered to prevent trial discontinuation through unethical or preventable means.

A second form of research waste occurs when results from completed RCTs are not published or their results made public in any form, although clinical trial investigators are obligated to disseminate the results of their research through publication or alternative means.^2,12^ Various reasons exist for nonpublication. It is widely reported that clinical trials have gone unpublished to prevent potentially unfavorable results from affecting business interests.^40^ In addition, studies with statistically significant results that support the research hypothesis are 3 times more likely to be published than studies with less favorable results.^17,21,43^ Thus, nonpublication of trial results can greatly limit our understanding of intervention efficacy, negatively affect patient care, and contribute to research waste.

Given the significant concerns associated with trial discontinuation and nonpublication, the primary objective of this cross-sectional analysis was to quantitatively evaluate the rates of discontinuation and nonpublication of RCTs involving patients with chronic pain.

## 2. Methods

### 2.1. Data source and collection

Before commencing any clinical trial, all U.S. investigators must register their trial on ClinicalTrials.gov—an online repository provided by the U.S. National Library of Medicine—and provide details regarding their primary objectives and continually update results as the trial progresses. When an RCT is registered, an individual national clinical trial number (NCT) is assigned to each entry and carried throughout the completion of the study. Using the advanced search function on ClinicalTrials.gov, we conducted a focused search of phase 3 and phase 4 registered RCTs regarding chronic pain on ClinicalTrials.gov. ClinicalTrials.gov additionally has an automated term-mapping capability, which was used to add several related terms to our search including pain, nonmalignant chronic pain, postoperative pain, neuropathic pain, fibromyalgia, and low back pain. Our search was deployed on October 5, 2020, and included all trials up to this date (ClinicalTrials.gov began recording trials in 2000). Trials were then identified and downloaded and formatted for analysis using Microsoft Excel, version 16.30 (Microsoft Corporation). Because this cross-sectional analysis consists of reviewing and synthesizing already published data, this study is not subject to ethical approval.^10^

### 2.2. Inclusion and exclusion criteria

Trials with an updated trial status of completed, suspended, terminated, withdrawn, or unknown were included in our sample. Trial status not yet recruiting, recruiting, enrolling by invitation, and active not recruiting were excluded. All studies were organized into 2 categories: completed, which included studies with the status of completed, and discontinued, which included studies with the status of terminated, withdrawn, unknown, or suspended. Trials published after October 1, 2017, were excluded to allow investigators 36 months from the date of trial completion for publication.^6,19,39^ Furthermore, RCTs had to be relevant to chronic pain and be a phase 3 or phase 4 trial. Trials that combined phase 2 and 3 were included in our sample. We elected to include phase 3 and phase 4 trials because they constitute the highest level of evidence, with the intent of subsequent publication.^13^ No limitations were placed on patient age or demographic.

### 2.3. Publication history

Search results are screened by registered title, condition, study design, and completion date. To identify whether the registered clinical trial published results regarding their experiment, we searched ClinicalTrials.gov for any linked publication. If no publication could be found, we then searched Google Scholar, Embase, and PubMed (which encompasses MEDLINE) for trial NCT, title, or authors to identify any publication. We defined publication in this study as any release of trial results in a peer-reviewed journal in the form of a complete article.

### 2.4. Contact information search

Trialists were contacted if no reason for discontinuation was listed or if no publication could be located from our systematic search. In an attempt to allocate this information, we contacted trialists to locate any possible publication or reason for discontinuation of trial. To identify contact information for the corresponding trialist, we searched ClinicalTrials.gov, institutional websites, PubMed-listed publications from the trial's corresponding author, and Google. If contact information was identified, a standardized message that included the trial's NCT was sent to identify missing information (Supplemental File 1, available at http://links.lww.com/PR9/A188). We followed the standardized contact information used with success in other studies attempting to contact authors.^6,19,39,41^ This method included a weekly attempt to contact for 3 consecutive weeks. Authors were deemed noncontactable if no response was received after 8 weeks or the message came back as undelivered. If we were unable to provide evidence or identify any publication through all avenues, we considered the trial to be unpublished.

### 2.5. Statistical analysis

Summary statistics represent the data as percentages and frequencies, with appropriate confidence intervals. A binary logistic regression was used to calculate adjusted and unadjusted odds ratios (uORs) for the association between trial characteristics (source of funding, intervention, and sample size) and completion (0 indicated discontinued and 1 completed) or publication status (0 indicated unpublished and 1 published). Statistics were run using STATA 15.1 (StataCorp, LLC, College Station, TX).

### 2.6. Trends of discontinued and unpublished trial data

Each discontinued and unpublished study was evaluated to determine if there was a trend in their available data. The data were classified as *trending positive* if there was a statistically significant result that was in favor of the treatment outcome, *trending negative* if the data were trending towards a statistically insignificant result that was unfavorable or the study showcased the treatment led to a large number of adverse effects, and *inconclusive* if the study was terminated due to reasons outside of the treatment modality, too small of a study population according to the trialist, or if the study had conflicting results that showed benefit and harm. If no results were available, the study was categorized accordingly.

## 3. Results

### 3.1. Study characteristics

**Figure 1.**
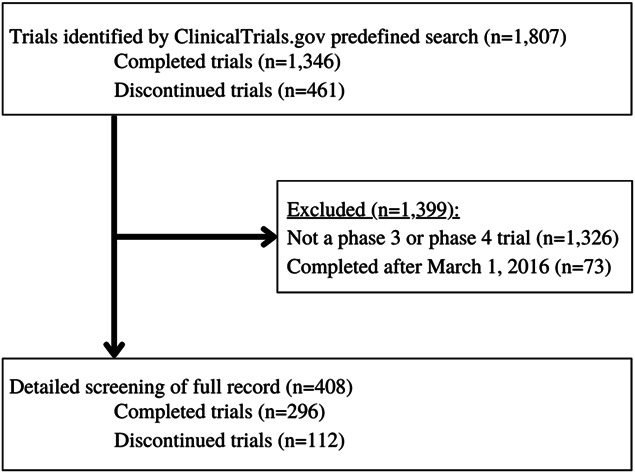
Process for including and excluding randomized controlled trials.

The initial search yielded 1807 returns related to chronic pain, with 1346 completed and 461 discontinued trials. After screening all of the returns against our inclusion criteria, our final analysis included 408 RCTs. Seventy-three trials were excluded owing to completion after October 1, 2017, and 1326 were excluded because they were not either phase 3 or 4 trials (Fig. 1). Of the 408 included RCTs, 245 (60.0%) were phase 3 RCTs and 163 (40.0%) were phase 4 RCTs. The included trials most frequently investigated pharmacologic (drug or biologic) interventions for chronic pain (321 of 408 trials [78.7%]). A total of 223 of the 408 trials (54.7%) were funded by the pharmaceutical industry. Table 1 provides further details regarding trial characteristics. The trial registration dates ranged from January 1, 2000, to October 1, 2017. The median number of study enrollments or participants was 120 patients (interquartile range, 43.25–305.0 patients, range, 0–3021 patients). There were 178 RCTs (43.6%) with a sample size less than 100, 226 (55.4%) with a sample size of at least 100 and 4 (0.01%) that did not report a sample size.

**Table 1:** Characteristics of completed and discontinued trials.

Characteristics	Completed N (72.5%)	Discontinued N (27.4%)	Total N (100%)
	(n = 296)	(n = 112)	(n = 408)
Intervention (n = 408)			
Medical device	18 (47.4%)	20 (52.6%)	38 (9.3%)
Pharmaceutical	243 (75.7%)	78 (24.3%)	321 (78.7%)
Behavioral	14 (93.3%)	1 (6.7%)	15 (3.7%)
Procedure	15 (57.7%)	11 (42.3%)	26 (6.4%)
Others	6 (75%)	2 (25%)	8 (1.9%)
Funding source (n = 408)			
Industry	174 (78.0%)	49 (22.0%)	223 (54.7%)
Federal	20 (64.5%)	11 (35.5%)	31 (7.6%)
Others*	101 (66.9%)	50 (33.1%)	151 (37.0%)
Mixed	1 (33.3%)	2 (66.7%)	3 (0.7%)
Enrollment (n = 408)			
<100	102 (57.3%)	76 (42.7%)	178 (43.6%)
≥100	192 (85.0%)	34 (15.0%)	226 (55.4%)
Not reported†	2 (50.0%)	2 (50.0%)	4 (1.0%)

* Others: private, hospitals or university, and nonprofit or for-profit organizations.

† Trials did not report participant enrollment, or the report was null.

### 3.2. Discontinuation of clinical trials

**Figure 2.**
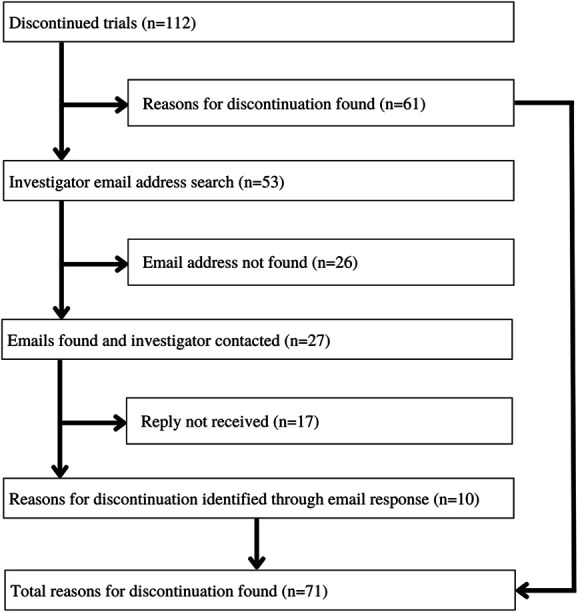
Randomized controlled trial discontinuation breakdown.

There were 408 included RCTs. A total of 296 (72.5%) of the trials were completed and 112 (27.5%) were discontinued. Of the 296 completed trials, 110 had results posted on ClinicalTrials.gov. Based on the 112 discontinued trials, 10,907 patients were recruited, which represents 11.9% (10,907 of 91,466) of the total study participants. Before any external communication, 53 of 112 (47.3%) did not provide reasons for trial discontinuation on ClinicalTrials.gov. Contact email addresses were identified for 27 of 53 discontinued trials (50.9%) that did not list a reason for trial discontinuation, and responses were received for 10 of 27 emails (37%). After collecting the email responses, a final analysis identified 71 of 112 trials (63.4%) providing a reason for trial discontinuation (Fig. 2). Reasons identified for discontinuation of trials provided from all sources included administrative recommendations (such as corporate reasons unrelated to safety and efficacy, changes in company strategy, positive preliminary results from other studies, or because of recommendation from the data monitoring committee) (41 of 71 trials [57.7%]), withdrawn funding or support from the trial sponsor (6 of 71 [8.5%]), and low accrual or insufficient recruitment (24 of 71 [33.8%]). In addition to providing reasons for trial discontinuation, email responses identified discontinued trials that were published but not found during a literature search or recorded on ClinicalTrials.gov. Of the 112 discontinued trials, 63 (56.2%) were phase 3 trials and 49 (43.8%) were phase 4 trials. The registration years ranged from January 1, 2000, to October 1, 2017, for discontinued trials, with no year having a considerably higher percentage of discontinued trials. The median number of study enrollment or participants in discontinued RCTs was 45.5 patients (interquartile range, 11.5–122.0 patients, range, 0–849 patients).

### 3.3. Nonpublication of clinical trials

Our initial search identified 88 unpublished completed trials (of 296 total, 29.7%). Seventy-six of the email addresses (86.4%) were identified, and the primary investigator was contacted. The remaining 12 (13.6%) email contacts were unknown. Each investigator was allotted 8 weeks for a response. Eighteen of the 76 emails (23.7%) resulted in a reply to our query, which identified 13 trials that were completed and published but not recorded on ClinicalTrials.gov or found during our literature search.

### 3.4. Overall publication status of completed and discontinued clinical trials

**Figure 3.**
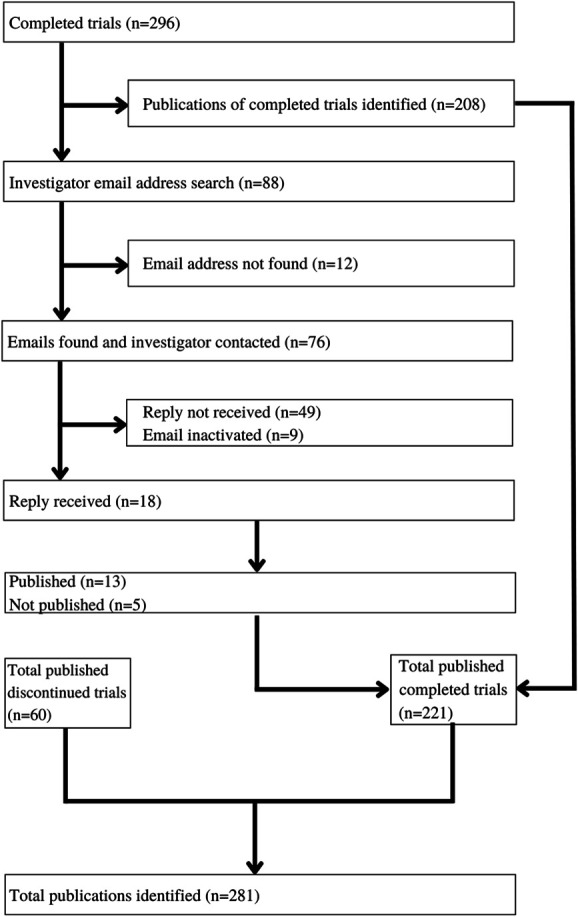
Randomized controlled trial publication breakdown.

After hand-searching through trial registries, MEDLINE, and email responses, our final analysis of the 408 RCTs included 281 trials (68.9%) that were published in a peer-reviewed journal and 127 trials (31.3%) that were unpublished. Eighty-three of the 127 unpublished trials (65.4%) were phase 3 trials, whereas the other 44 (34.6%) were phase 4 trials. Fifty-nine of 112 discontinued trials (52.7%) reached publication. In addition, 221 of 296 completed trials (74.7%) were published (Fig. 3). The registration years of unpublished trials ranged from January 1, 2000, to October 1, 2017, with no year having a considerably higher percentage of unpublished trials. Table 2 shows trial characteristics according to the publication status.

**Table 2:** Characteristics of published and unpublished clinical trials.

Characteristics	Published N (68.6%)	Unpublished N (31.4%)	Total N (100%)
	(n = 281)	(n = 127)	(n = 408)
Intervention (n = 408)			
Medical device	16 (42.1%)	22 (57.9%)	38 (9.3%)
Pharmaceutical	225 (70.1%)	96 (29.9%)	321 (78.7%)
Behavioral	13 (86.7%)	2 (13.3%)	15 (3.7%)
Procedure	20 (76.9%)	6 (23.1%)	26 (6.4%)
Others	7 (87.5%)	1 (12.5%)	8 (1.9%)
Funding source (n = 408)			
Industry	139 (62.3%)	84 (37.7%)	223 (54.7%)
Federal	25 (80.6%)	6 (19.4%)	31 (7.6%)
Others*	115 (76.2%)	36 (23.8%)	151 (37.0%)
Mixed	2 (66.7%)	1 (33.3%)	3 (0.7%)
Enrollment (n = 408)			
<100	110 (61.8%)	68 (38.2%)	178 (43.6%)
≥100	169 (74.8%)	57 (25.2%)	226 (55.4%)
Not reported†	2 (50.0%)	2 (50.0%)	4 (1.0%)

* Others: private, hospitals or university, and nonprofit or for-profit organizations.

† Trials did not report participant enrollment, or the report was null.

### 3.5. Logistic regression

Of the 408 RCTs, a total of 91,466 participants were recruited. In the trials that did not reach publication, there were 18,799 participants (calculated from actual or target registration data in ClinicalTrials.gov).

Clinical trials funded by other sponsors (private, hospitals/university, or nonprofit or for-profit organizations) were more likely to reach publication than industry-funded clinical trials (uOR 1.86 [95% CI, 1.18–2.95]; odds ratio (aOR) 3.01 [95% CI, 1.76–5.14]). Pharmaceutical intervention clinical trials were more likely to be completed and more likely to reach publication, than medical device intervention clinical trials (uOR 3.46 [95% CI, 1.74–6.87]; aOR 2.12 [95% CI, 1.05–4.33], and uOR 3.22 [95% CI, 1.62–6.40]; aOR 2.55 [95% CI, 1.23–5.29], respectively). Additional associations with discontinued or unpublished RCTs and intervention type, funding source, or sample size can be found in Table 3.

**Table 3:** Logistic regression of trial discontinuation and nonpublication.*

Characteristics	No. discontinued (27.4%), n = 112			No. unpublished (31.4%), n = 127		
	No. (%)	uOR [95% CI]	aOR [95% CI]	No. (%)	uOR [95% CI]	aOR [95% CI]
Intervention						
Medical device	20 (52.6%)	1 [reference]		22 (57.9%)	1 [reference]	
Pharmaceutical	78 (24.3%)	3.46 [1.74–6.87]	2.12 [1.05–4.33]	96 (29.9%)	3.22 [1.62–6.40]	2.55 [1.23–5.29]
Behavioral	1 (6.7%)	15.56 [1.86–130.42]	11.61 [1.33–101.00]	2 (13.3%)	8.94 [1.77–45.25]	4.54 [0.86–23.90]
Procedure	11 (42.3%)	1.52 [0.55–4.14]	1.02 [0.34–3.10]	6 (23.1%)	4.58 [1.50–14.00]	2.13 [0.65–6.99]
Others	2 (25%)	3.33 [0.69–18.66]	2.42 [0.42–14.08]	1 (12.5%)	4.13 [0.73–23.15]	2.35 [0.40–13.93]
Funding source						
Industry	49 (22.0%)	1 [reference]		84 (37.7%)	1 [reference]	
Federal	11 (35.5%)	0.51 [0.23–1.14]	0.80 [0.33–1.96]	6 (19.4%)	2.52 [0.99–6.39]	3.35 [1.23–9.04]
Others†	50 (33.1%)	0.57 [0.36–0.90]	1.08 [0.63–4.66]	36 (23.8%)	1.86 [1.18–2.95]	3.01 [1.76–5.14]
Mixed	2 (66.7%)	0.14 [0.13–1.59]	0.40 [0.03–4.66]	1 (33.3%)	1.21 [0.11–13.53]	2.93 [0.24–35.07]
Enrollment						
Trial participants sample size (continuous)	N/A	1.00 [1.00–1.01]	1.00 [1.00–1.01]	N/A	1.00 [1.00–1.00]	1.00 [1.00–1.00]

* Logistic regression adjusted for funding source, intervention, and sample size.

† Others: private, hospitals or university, and nonprofit or for-profit organizations.

uOR, unadjusted odds ratio; aOR, adjusted odds ratio; CI, confidence interval.

### 3.6. Trends of discontinued and unpublished trial data

We further evaluated the trends of the available data on ClinicalTrials.gov. We assessed 112 discontinued trial data and 127 unpublished trial data to determine if the data were trending positive, trending negative, or inconclusive at the time the trial was discontinued or ended nonpublished. Of the discontinued trials, 2 of 112 (1.8%) were trending positively, 5 of 112 (4.5%) were trending negatively, 15 of 112 (13.4%) had inconclusive data, and 90 of 112 (80.4%) did not have data available. Of the unpublished trials, 3 of 127 (2.4%) were trending positively, 19 of 127 (15.0%) were trending negatively, 4 of 127 (3.1%) had inconclusive data, and 101 of 127 (79.5%) did not have data available. The results of the trends can be found in Table 4.

**Table 4:** Trends of discontinued and unpublished trial data.

	Trending positive	Trending negative	Inconclusive	No study data available	Total studies
Discontinued	2	5	15	90	112
Unpublished	3	19	4	101	127

## 4. Discussion

Our study demonstrated that approximately one-fourth of chronic pain RCTs was discontinued, and these trials included over 10,000 participants. Slightly more than half of the discontinued trials on ClinicalTrials.gov listed reasons for discontinuation. Interestingly, we found that *medical devices* were the intervention with the highest rates for both discontinued (Table 1) and unpublished trials (Table 2). After contacting trialists, more than one-third of trials remained with unknown reasons for discontinuation. This information was unable to be obtained due to a lack of response to our standardized email or no email address provided or undeliverable email address. This rate of discontinuation is significantly higher than what was found in similar studies investigating head and neck cancers, osteoarthritis, and alcohol use disorder.^15,19,39^ As previously discussed, the considerations for why chronic pain research has such higher rates of discontinuation and unpublished literature compared with other fields is likely due to not wanting the public to see unfavorable results or insignificant findings in a multibillion dollar industry that affects so many patients. Randomized controlled trials can subject participants to harmful therapies which may lead to adverse events, but trial discontinuation and nonpublication inhibits awareness of such results—increasing the likelihood of harming additional patients while wasting valuable resources. Therefore, every effort should be made to optimize quality, improve conduct, and eliminate research waste of chronic pain clinical trial data. Here, we will discuss how chronic pain RCTs are essential in determining quality care for patients, demonstrate how discontinued and unpublished trials impede progress, and suggest methods to enhance the efficacy of future chronic pain RCTs.

Approximately half of the one-fourth of discontinued chronic pain RCTs was discontinued without reason or explanation justifying its suspension. This statistic is concerning because most participant withdrawal from chronic pain RCTs is attributed to intolerable adverse events or lack of efficacy.^23,25,29–31^ As such, a trial examining the efficacy of tapentadol, a centrally acting analgesic prescribed to alleviate pain caused by osteoarthritis, found that nearly one-fifth of participants complained of intolerable adverse events, warranting trial discontinuation.^23^ Our sample alone indicates a possibility of nearly 20,000 participants being exposed to potentially damaging interventions or harmful drugs—all without record or documentation. Results from discontinued trials, along with its missing data, are important clinical outcomes, and, thus, parameters need to be set in place to reduce research waste and help prevent adverse patient outcomes.^28,34^

Furthermore, nearly one-third of the trials in our sample were unpublished. This statistic is similar to the trend reported by Korevaar and Hooft where between one-fourth and one-half of all clinical trial data go unpublished.^21^ Unpublished trial data are concerning because when research is inaccessible, billions of dollars are wasted, bias is introduced, and patient care declines.^5^ One recent metaepidemiological study evaluating publication bias across multiple medical specialities found that trials on treatment effects regarding chronic pain were more likely to report statistically significant results leading to a higher probability of the introduction of publication bias.^38^ If trial results remain unpublished, future researchers could encounter the same preventable errors that took place in earlier RCTs that could have been corrected and learned from had results been accessible. Mandatory publication of RCT results has frequently been mentioned as the most effective way of reducing publication bias, particularly when trials have negative or insignificant results that the researchers and their funders may not want the public to have access to.^4^ Further investigation is warranted to identify the best ways to disseminate RCT results that are unpublished to ensure participant safety, gain knowledge, and reduce publication bias.

Because of our findings, we recommend that efforts be made to improve trial completion and publication. Several studies express the importance of a lead investigator devoted to the completion of a clinical trial through a focus on crucial aspects such as maximizing recruitment and minimizing participant dropout.^7,16,42^ Evidence continues to suggest that the presence of an enthusiastic lead investigator—who provides quality leadership to a clinical trial—substantially minimizes participant adverse outcomes leading to greater trial completion and publication.^11^ Nipp et al.^33^ suggests that an abundance of well-trained trial staff results in a higher rate of trial completion due to better accrual and the retention of trial participants. In addition, for completed trials, publication of their results must remain a priority. New avenues for publication now exist, such as preprint servers, and these mechanisms may be used to publish results from completed trials. We do caution that preprint servers are controversial, as articles deposited on preprint servers have not been peer reviewed. Thus, this avenue warrants more exploration before larger scale efforts are made for their use in disseminating clinical trial results.

Large databases or working groups that enable data sharing for chronic pain studies could improve publication rates. One such example includes the work of the Enhancing Neuroimaging Genetics through Meta-Analysis Consortium. Enhancing Neuroimaging Genetics through Meta-Analysis is a collaborative network of researchers who are combining efforts to analyze neuroimaging data from around the world. Their mission is to share ideas, algorithms, data, and information on promising findings and methods to facilitate training on key methods and emerging directions in imaging genetics.^1^ This type of sharing platform has proven to be beneficial in the field of neuroimaging genetics. Similarly structured consortiums dedicated to chronic pain studies could lead to promising results.

### 4.1. Limitations

Our study is not without limitations. We acknowledge that although our search strategy was comprehensive, it is possible that we did not capture all RCTs pertaining to chronic pain. In addition, generalizability to all clinical trial types may not be possible as our sample was limited to include only Phase 3 and 4 RCTs. Although we made an attempt to contact authors, not all studies contained a working email address for all authors within the trial registry. This further demonstrates the necessity of communicating changes to contact information in the trial registry in order for the public and researchers to be able to contact the authors to improve the availability of results of unpublished trials.

## 5. Conclusion

Our study demonstrates that improvement is essential to reduce the number of unpublished and discontinued RCTs in chronic pain. Clinical trial information is crucial for providers to be able to provide evidence-based medicine in the treatment of chronic pain. When RCTs are discontinued or go unpublished, resources are wasted and the health of the participants may be compromised. Every effort should be made by researchers and funding organizations to improve the quality and execution of chronic pain clinical trials to minimize research waste and aid in guiding quality patient care.

## Disclosures

The authors have no conflict of interest to declare.

S. M. Jacobsen has no financial disclosures. T. Moore has no financial disclosures. A. Douglas has no financial disclosures. D. Lester has no financial disclosures. A. L. Johnson has no financial disclosures. M. Vassar is funded through the U.S. Department of Health and Human Services Office of Research Integrity and the Oklahoma Center for the Advancement of Science and Technology.

## Appendix A. Supplemental digital content

Supplemental digital content associated with this article can be found online at http://links.lww.com/PR9/A188.
