# Supplementary material for: Discontinuation and nonpublication analysis of chronic pain randomized controlled trials
Source: Pain Rep. 2023 Apr 4;8(3):e1069. doi: 10.1097/PR9.0000000000001069 (PMC10079346; doi:10.1097/PR9.0000000000001069)
Supplement: Supplementary file 1 [file painreports-8-e1069-s001.docx]

**Supplemental File 1.** Standardized email used to evaluate rates of publication of completed trials.

Trial Discontinuation Study - NCT##############

We are conducting a study to evaluate the rates of publication of trials registered as “Completed” on clinicaltrials.gov. We identified you as the primary investigator or trial contact on a trial entitled, “___________” (NCT##############). To make our study results as complete as possible, we would like to know whether your completed trial has been published. In the event that your trial was not published, we would also like to know the reason for non-publication. We would appreciate your response to the following questions.

Was this “Completed” trial published? Yes No

If so, please provide the reference information, including authors’ names, title, journal, and year of publication or other information necessary to locate your published trial.

If **NOT** published, please provide the **reason or reasons** for non-publication from the list below:

1. Lack of time
2. Low priority
3. Lack of funding
4. Lack of manpower
5. Study incomplete or ongoing
6. Publication not an aim
7. Author/Co-author problems
8. Poor study quality or design
9. Fear/Expectation of journal rejection
10. Results not important enough
11. Results negative
12. Similar findings published previously
13. Sponsor/Funder problems
14. Publication not permitted by sponsor
15. In preparation or under review
16. Initial submission rejected, no intention of resubmission
17. Inadequate sample size/recruitment problems
18. Study terminated due to adverse events/toxicity
19. Other

Thank you for your assistance in our study,
 [email signature]
